# Reducing Water Sensitivity of Chitosan Biocomposite Films Using Gliadin Particles Made by In Situ Method

**DOI:** 10.3390/polym9110583

**Published:** 2017-11-06

**Authors:** Dajian Huang, Zonghong Ma, Zhuo Zhang, Qiling Quan

**Affiliations:** School of Mechanic and Electronic Engineering, Lanzhou Jiaotong University, Lanzhou 730070, China; mzh316718@163.com (Z.M.); zhang1466rose@163.com (Z.Z.); quanql0329@163.com (Q.Q.)

**Keywords:** chitosan, gliadin, in situ method, water resistance, mechanical properties, optical properties

## Abstract

In order to sustain rapid expansion in the field of biocomposites, it is necessary to develop novel fillers that are biodegradable, and easy to disperse and obtain. In this work, gliadin particles (GPs) fabricated through an in situ method have been reported as fillers for creating chitosan (CS)-based biocomposite films. In general, the particles tend to agglomerate in the polymer matrix at high loading (approximately >10%) in the biopolymer/particles composites prepared by the traditional solution-blending method. However, the micrographs of biocomposites confirmed that the GPs are well dispersed in the CS matrix in all CS/GPs composites even at a high loading of 30% in this study. It was found that the GPs could improve the mechanical properties of the biocomposites. In addition, the results of moisture uptake and solubility in water of biocomposites showed that water resistance of biocomposites was enhanced by the introduction of GPs. These results suggested that GPs fabricated through an in situ method could be a good candidate for use in biopolymer-based composites.

## 1. Introduction

Biopolymers and their biodegradable films have been extensively developed in response to both environmental concerns regarding non-biodegradable materials and the energy crisis in the past decades [1,2,3,4,5,6]. Among these developments, carbohydrates, such as chitosan (CS), have attracted scientific and industrial interest in widely differing fields due to their unique properties, such as renewablility, biodegradability, and excellent film-forming capability [7,8,9,10,11]. Despite these advantages, pure CS-based films also show some advantages such as poor mechanical properties, high brittleness and high water sensitivity, thereby limiting their extensive applications [12,13].

An effective strategy that enhances the properties of pure CS-based materials is the addition of fillers into the CS matrix to create composites [14,15,16,17,18]. However, fillers tend to aggregate together through strong Van der Waals interactions, which hinder the dispersion of fillers in the CS matrix and decrease the properties of composites with high loading [11,19,20]. In addition, chemicals are usually used in preparing fillers, thereby causing environmental problems [17,21,22,23,24]. Thus, a significant demand for fillers that could disperse well in the CS matrix and are prepared by a green method is needed. Gliadin (Gd), the major storage protein in wheat seeds, has been considered as a resourceful food biopolymer because of its special solubility and strong hydrophobicity [25,26]. Gd can self-assemble to form particles in water or polysaccharide solution by an antisolvent technique because of its special tertiary structures [27,28]. These Gd particles (GPs)/polysaccharide mixtures may possibly form polysaccharide/GPs biocomposites with uniform dispersion, through the use of an in situ method in the polysaccharide matrix after solvent evaporation. Thus, GPs may be a good candidate as filler to reduce the water sensitivity of CS film because of its high dispersion and unique hydrophobicity.

This study aims to develop biodegradable films based on CS, which is reinforced with various amounts of GPs that are prepared via the in situ method. The dispersion of GPs in the CS matrix, and the water resistance and mechanical properties of biocomposite films were investigated to establish a structure–property correlation between the filler and the matrix.

## 2. Materials and Methods 

CS from Sinopharm Chemical Reagent Co., Ltd., (Shanghai, China) with 80% degree of deacetylation was used in this study. Analytical grade sorbitol and ethanol were commercially available, and used without further purification. Gluten was kindly provided by Shandong Rongxiang Food Additives Co., Ltd. (Rongcheng, China).

The Gd powder was extracted following the procedure explained in a previous paper with slight modifications [25]. Gluten powder dispersions were prepared by adding 50 g gluten powder in 500 mL of ethanol/water binary solution (70/30 *v/v*) under magnetic agitation at 200 rpm for 6 h at room temperature. Then, the obtained mixture was centrifuged for 15 min. The supernatant containing the gliadin-rich fraction was collected and dialyzed against deionized water for 48 h. After that, the dialysate was lyophilized to produce Gd powders.

The biocomposite films were prepared using the solution-casting method. CS (2%) solutions were prepared by dissolving CS in aqueous acetic acid solution (1%) at room temperature and stirring for 24 h. Subsequently, sorbitol was added into the system at 30% loadings with respect to CS weight and mixture was stirred for 4 h to obtain homogeneous solutions. The Gd solution (1%) was prepared by dissolving Gd in 70% (*V/V*) ethanol in water solution. The Gd solutions were dropped into the CS solution using a 10.0 mL syringe through a 1.7 mm diameter needle to form CS/Gd suspensions with a mechanical stirring at 500 rpm/min and 0%, 5%, 10%, 20%, and 30% (*w*/*w*) Gd loadings with respect to CS weight. Then, the homogeneous solutions were poured into 9 cm diameter polycarbonate petri dishes and evaporated in atmosphere for 72 h. The resulting films were dried in a vacuum oven (at 70 °C) for 6 h to remove traces of the solvents. The CS/Gd biocomposite films were stored in a desiccator in preparation for further treatment.

In order to study the morphology of films, field emission scanning electron microscopic (FESEM) was measured by a JEOL JSM-6701F (Japan Electron Optics Limited, Tokyo, Japan). The CS and CS/GPs films were immersed in liquid nitrogen and cryo-fractured manually. The films were coated with gold prior to observation. Light transmission of films was studied at selected wavelengths between 200 and 800 nm by a UV–Vis spectrophotometer (N4, INESA (Group) Co., Ltd., Shanghai, China). 

Tensile strength and elongation at break of films were measured using an AG-IS material testing machine (Shimadzu Co., Ltd., Kyoto, Japan) equipped with a 200 N load cell at room temperature with gauge length of 40 mm and crosshead speed of 10 mm/min. The moisture uptake (*MU*) of the samples was determined according to the following method. The films (2 cm × 3 cm) were dried at 70 °C for 6 h in an oven until a constant mass was reached, and placed inside a desiccators maintained at 75% relative humidity using NaCl saturated solution until equilibrium was reached. After being weighted (*m*_0_), the samples were dried at 70 °C in an oven to reach constant mass and weighed again (*m*_1_). The dried films were then immersed in 50 mL of distilled water for 24 h. Subsequently, the films were taken out of the water and dried at 70 °C to constant weight (*m*_2_). 

*MU* was calculated by using the following equation:

*MU* = [*m*_1_ − *m*_0_]/*m*_0_ × 100% (1)

Solubility in water (*WS*) was calculated using the following equation:

*WS* = [*m*_1_ − *m*_2_]/*m*_1_ × 100% (2)

Averages and standard deviations from at least three measurements of each sample were reported in this study. Student’s *t*-test was used for analysis of the test results at the significance level of *p*-value < 0.05.

## 3. Results

**Figure 1:**
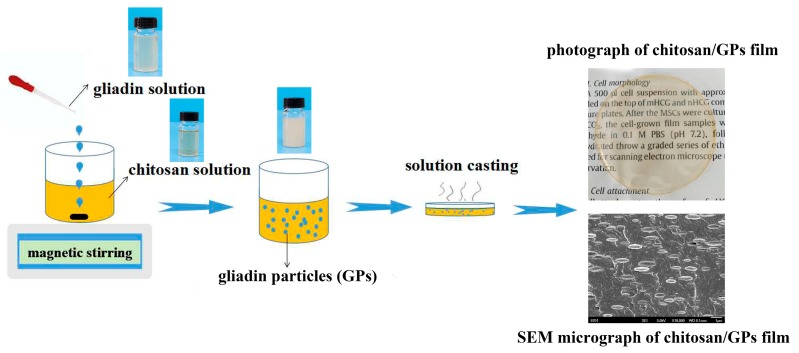
Schematic of fabrication mechanism of chitosan/gliadin particles (CS/GPs) biocomposites.

Figure 1 shows the fabrication process of CS/GPs biocomposites and the photographs of CS, Gd, and CS/Gd solutions, which were all placed at atmosphere for 15 days. The color of the CS and Gd solutions is slightly yellow. The colloidal solution of CS/Gd composite was formed because of the formation of Gd particles (GPs). GPs have been produced through antisolvent procedures in the previous studies [27,28]. Antisolvent process is an approach to produce colloidal particles through supersaturation, that is, by mixing a solvent with an antisolvent. In this study, we prepared GPs through antisolvent procedures using CS solutions as antisolvent and obtained a series of colloidal solutions of CS/GPs biocomposites. No agglomerates were found in composite suspensions with 10 wt % GPs loadings after 15 days, thereby indicating that GPs can disperse well in the CS solution. The GPs may be wrapped by CS molecules, which can reduce the hydrophobic attraction between the GPs.

**Figure 2:**
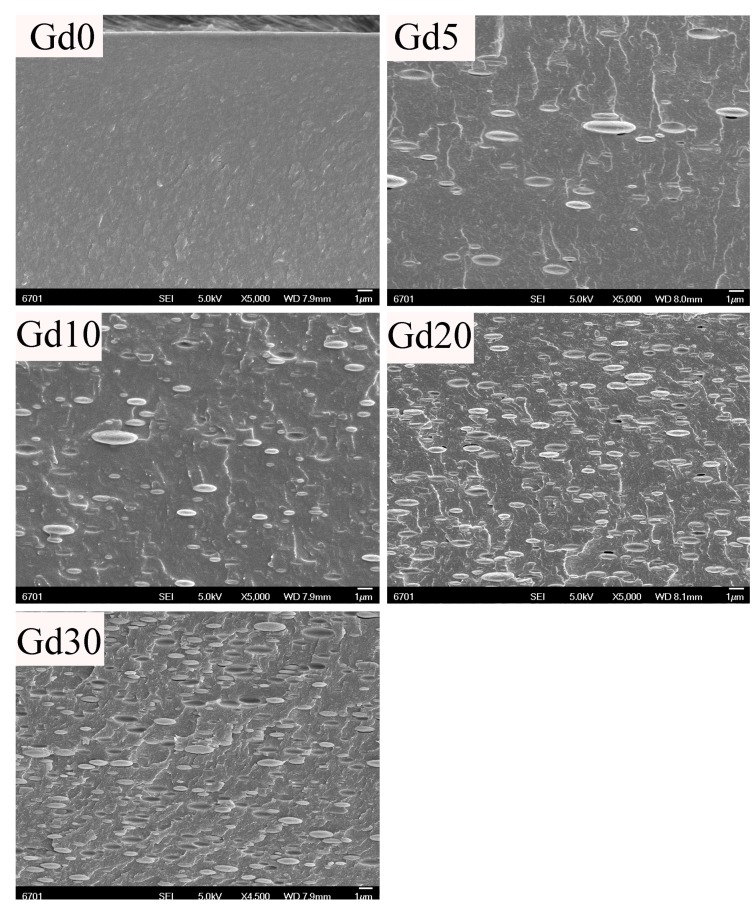
SEM micrographs of of CS/GPs composites with various amounts of added GPs.

The dispersion of fillers is a critical factor in determining the properties of biocomposites [29]. FESEM was used to study the dispersion of GPs in biocomposites. Figure 2 shows the corresponding images for neat CS and biocomposites. The fractured surface morphology of neat CS film (Gd0) is nonporous, smooth, and membranous without any microstructures. After the incorporation of Gd, GPs can be observed from the four types of CS/GPs biocomposites. GNs exhibit an oval-shaped appearance and their quantity gradually increased on the fractured surface with an increased GPs content from 5% to 30%. In general, the particles tend to agglomerate in the polymer matrix at high loading (approximately >10%) [30]. However, the GPs are well dispersed in the CS matrix in all CS/GPs composites even at high loading of 30% in this study. This behavior can be attributed to the fact that the CS acted as an effective stabilizer to prevent the aggregation of GPs in solutions. Similar results were reported by Zhang et al. [31], suggesting that CS can hinder the aggregation of zein nanoparticles in solutions. In addition, the surfaces of GPs are covered by CS, indicating good adhesion between GPs and the CS matrix. 

**Figure 3:**
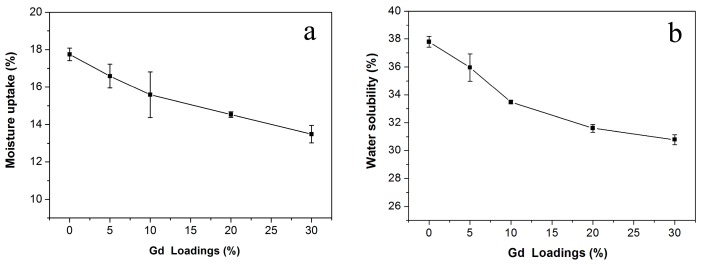
Moisture uptake (*MU*) (**a**) and solubility in water (*WS*) (**b**) of CS/GPs biocomposites with various amounts of added GPs.

Moisture content is a key factor in determining the application of biopolymer films. Pure CS film displays high moisture absorption because of its hydrophilic character, thereby resulting in films with relatively poor mechanical properties at high humidity [32]. Figure 3a shows the *MU* of CS/GPs biocomposites with various loadings. The value of *MU* of pure Gd film is 10.81%. The *MU* of CS/GPs biocomposites was lower than that of neat CS film and gradually decreased with an increase the GPs content from 0 to 30%. The reason could be that the surface of GPs is more hydrophobic than the surface of the CS matrix, and consequently, water has decreased affinity for the CS/GPs composites. In addition, the GPs in the CS matrix produce a tortuous pathway and a diminution of the length of free way for *MU*. *WS* is an important property of food packaging films due to their using as protective layers on food. Figure 3b shows the *WS* of neat CS films in distilled water was approximately 38%, which was higher than that of CS/GPs composites with GPs. The value of *WS* of pure Gd film is 7.18%. This result indicates that the incorporation of GPs reduced the *WS* of CS-based films. This observation is in accordance with the *MU* results, which can also be attributed to the more hydrophobic nature of GPs than the CS. Moreover, the GPs can act as a barrier and can reduce efficient contact between CS and water molecules.

**Figure 4:**
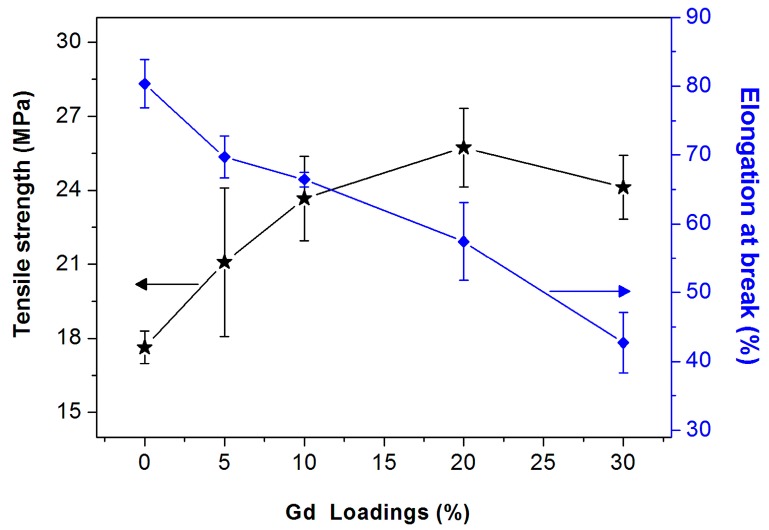
Tensile strength and elongation at break of CS/GPs composites with various amounts of added GPs.

Figure 4 shows the tensile strength (TS) and elongation at break (EB) of the CS/GPs biocomposites as a function of GPs loading. The mechanical behavior of CS/GNs is significantly influenced by the introduction GPs into the CS matrix. As expected from other studies on biopolymer films reinforced by alcohol-soluble protein nanoparticles [33,34], significant increase can be observed in TS because of the addition of Gd. Meanwhile, a decrease was observed in EB with the addition of GPs. The increasing in TS is related to the interfacial interaction between GPs and CS matrix. In addition, water could act as a plasticizer for the biopolymer film. Thus, the high moisture content of films would result in low TS. After the addition of GPs, the *MU* of CS/GPs decrease (*p* < 0.05), which would improve the TS of CS/GPs and decrease the EB. The reason for the slightly decreased TS of composites with higher GPs content (30%) is that more GPs destroy the continuity of the CS matrix.

**Figure 5:**
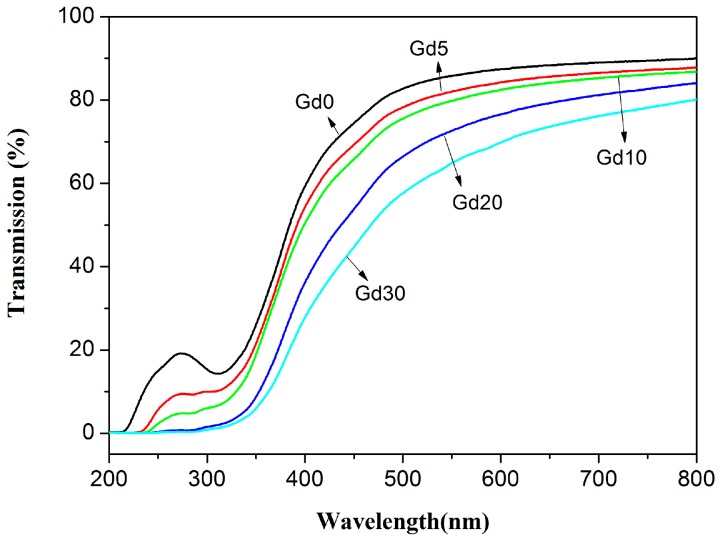
Optical properties of neat CS film and CS/GPs biocomposites.

The light transmittance of the neat CS and CS/GPs biocomposites was represented in Figure 5. It can be observed that the light transmittance of CS/GPs biocomposites are affected in the presence of the GPs, and the light transmittance decreases slightly with the increase in the content of GPs. However, all CS/GPs biocomposites show above 70% transparency at a wavelength of 800 nm. This behavior may due to the GPs being dispersed well in the CS matrix.

## 4. Conclusions

In conclusion, we have reported a convenient and green method to prepare CS/GPs mixtures. The resulting mixtures are homogeneous and exhibit long-term stability. After solvent evaporation, CS/GPs biocomposite films with uniform dispersion of GPs were successfully fabricated. The GPs show promising reinforcing efficiency in the mechanical behavior and water resistance of the CS/GPs biocomposite films, which can be ascribed to the uniform dispersion of GPs in the CS matrix and strong hydrophobicity of GPs. In addition, the introduction of GPs hardly affects the light transmittance of obtained biocomposites in the range of the visible light regions. We hope to be able to extend this procedure to the preparation of a range of GPs biocomposites based on the other biomacromolecule such as alginate, cellulose, gelatin etc. Therefore, we believe that this approach would provide an effective method to design and fabricate composite materials used in the fields of food packaging, and medicine with green and renewable raw materials.
